# Supplementary material for: Rehabilitation of brachial plexus injury in contact sport: Where are the data that underpin clinical management? A scoping review
Source: PLoS One. 2024 Jun 24;19(6):e0298317. doi: 10.1371/journal.pone.0298317 (PMC11195970; doi:10.1371/journal.pone.0298317)
Supplement: S1 File — (DOCX) [file pone.0298317.s002.docx]

**SUPPLEMENTAL DATA**

**S2A table: *Study design framework***

| **Design** | **Population** | **Concept** | **Context** |
| --- | --- | --- | --- |
| 1. All information sources and types to be considered. 2. Full text only 3. Available in English language 4. All publication years to be considered 5. Published peer-reviewed primary research articles | 1. Contact sport athletes who have sustained a brachial plexus injury during sports 2. Include worldwide population 3. Athletes of all ages. 4. Exclude non-sports related brachial plexus injury. | 1. Primary data research on management strategies for brachial plexus injuries. 2. Including injury assessment, diagnostics and return to play guidance. 3. Include studies where participants underwent surgical procedures for their brachial plexus injury. | 1. Contact and collision athletes participating in professional and amateur sports. 2. Pitch-side and medical/ physiotherapy management of their brachial plexus injury. 3. Rehabilitation protocols and return to play clearance, and pathways. |

**S2B file: *Search strategy:***

Search 1- “Brachial Plexus+/IN” OR “Brachial Plexus Neuropathies”.

Search 2- TI (“Brachial Plexus Injury” OR Stinger OR Stingers OR “Brachial Plexopathy” OR Burner) OR AB (“Brachial Plexus Injury” OR Stinger OR Stingers OR “Brachial Plexopathy” OR Burner).

Search 3- Search 1 OR Search 2.

Search 4- “Volleyball” OR “Wrestling” OR “Team Sports” OR “Soccer” OR “Rugby” OR “Football” OR “Hockey” OR “Basketball”.

Search 5- TI (Collision N2 Sport OR Collision N2 Athletes OR Contact N2 Sport OR Rugby OR Football OR Soccer OR Wrestling OR Volleyball OR Basketball OR Hockey OR Team N2 Sport*) OR AB (Collision N2 Sport OR Collision N2 Athletes OR Contact N2 Sport OR Rugby OR Football OR Soccer OR Wrestling OR Volleyball OR Basketball OR Hockey OR Team N2 Sport*).

Search 6- Search 4 OR Search 5.

Search 7- Rehabilitation OR “Physical Therapy” OR “Return To Play”.

Search 8- TI (Return* N2 Play OR Return N2 Sport* OR Rehabilitation OR Medical N2 Management”) OR AB (Return* N2 Play OR Return N2 Sport* OR Rehabilitation OR Medical N2 Management).

Search 9- Search 7 OR Search 8.

Search 10- Search 3 AND Search 6 AND Search 9.
