# Supplementary material for: Rehabilitation of brachial plexus injury in contact sport: Where are the data that underpin clinical management? A scoping review
Source: PLoS One. 2024 Jun 24;19(6):e0298317. doi: 10.1371/journal.pone.0298317 (PMC11195970; doi:10.1371/journal.pone.0298317)
Supplement: Attachment — Submitted filename: Review report.docx [file pone.0298317.s003.docx]

Review report

Dear authors

I have thoroughly reviewed your article on addressing deficiencies in the rehabilitation and clinical management of brachial plexus injuries in contact sports. I appreciate your efforts in this specific field. It is well-known that the incidence and severity of BPI varies based on the sport played. Depending on the degree of the injury's damage, the rehabilitation procedure varies. Many athletes return to sport without requiring postgame treatment.

Although the title of your study seems to be the rehabilitation of BPI, when I read the abstract and the entire article, I saw that your primary goal is to draw conclusions about the entire rehabilitation process and determine the criteria for return to sports. Despite the tight relationship between these two issues, they are distinct and must be addressed separately. If determining the clinical management and rehabilitation process is the primary objective, the chosen keywords are insufficiently specific. Because, on reviewing the entire article, I noticed that you included physiotherapy applications among the treatment methods. The study protocol that includes keywords also incorporates physiotherapy practices as keywords. Additionally, numerous other terms were used during the search. This indicates that the decision regarding keyword selection was inadequate. If the keywords are determined and chosen appropriately, you will be able to access more studies such as . S Reid et al.‘’ [Brachial plexus injuries—report of two cases presenting to a sports medicine practice](https://www.sciencedirect.com/science/article/pii/S1466853X02901227)’’.

In the article's introduction, injuries are categorized as severe or moderate. What do the terms severe and moderate mean? What is the frequency of these types of injuries? Even if it is in separate sports branches, it is necessary to mention it in order to inform the reader. Expressions such as uncommon and frequent provide insufficient information to the reader.

In paragraphs 79 and 80, is the phrase "In sports settings, BPIs are common enough for the slang terms'stinger' and 'burner' to be used?" a unique thought of the authors?

Writing the introduction section in a more comprehensible, fluent and holistic manner will keep the reader's interest in the article alive.

In the imaging, diagnosis, and surgical interventions section, the RTP abbreviation was used without its meaning being specified. Similarly, table abbreviations should also be defined.

Considering all of these circumstances

1- New and accurate keywords must be selected.

2- The purpose of the investigation must be determined thoroughly.

3- The introduction section should be written more clearly, fluently, and comprehensively.

4- The results and discussion sections should be rewritten based on the analysis of newly accessible articles.

I look forward to reviewing your article once these significant corrections have been made.
