# Supplementary material for: Rehabilitation of brachial plexus injury in contact sport: Where are the data that underpin clinical management? A scoping review
Source: PLoS One. 2024 Jun 24;19(6):e0298317. doi: 10.1371/journal.pone.0298317 (PMC11195970; doi:10.1371/journal.pone.0298317)
Supplement: Attachment — Submitted filename: BPI rsponse to reviewers_02-12-23.docx [file pone.0298317.s004.docx]

**Response to reviewers**

Dear Dr Akaras and editorial team,

Please find enclosed our revised manuscript entitled “Rehabilitation of brachial plexus injury in contact sport: Where are the data that underpin clinical management? A scoping review” which we re-submit for consideration of publication in PLOS ONE.

We are grateful to the reviewers for their comments and points for improvement. We are somewhat reassured that the issues raised, primarily related to the concise nature of our introduction section and have revised this section accordingly. We respond to the feedback in point-by-point fashion below. The manuscript has been clearly enhanced by the journals peer-review process.

Please note that in addressing the reviewer 2’s concerns, we reviewed the original search parameters and the returned documents, and then re-evaluated these with the assistance of an additional reviewer, Dr Mike Leavitt. Due to the degree of work carried out, we invited Dr Leavitt to join us as an author on the revised submission. We fully acknowledge the need for explicit clarity here to establish the appropriateness of additional authors joining a submission at the revision stage. We attest that Dr Leavitt has contributed to data capture and interpretation, and in the writing of the revised document, and approval for submission, and as such, we believe he fully meets the ICMJE criteria for authorship and propose his addition to the team to be entirely appropriate, and indeed necessary, to reflect the work he has carried out on this manuscript.

This remains a scoping review of the data that underpins and informs clinical management of patients with brachial plexus injuries, of which there are limited data and our discussion reflects this. As such, we feel this is an important finding and we feel that PLOS ONE is the perfect home for this manuscript.

Best wishes

Author team

**Editor’s comments to authors:**

Thank you for submitting your manuscript to PLOS ONE. After careful consideration, we feel that it has merit but does not fully meet PLOS ONE’s publication criteria as it currently stands. Therefore, we invite you to submit a revised version of the manuscript that addresses the points raised during the review process.

**AUTHORS:**

Thank you for the chance to respond to these two reviews. They clearly agree that our review is relevant and highlights an important paucity of data in the literature to support clinical management.

While the feedback has allowed us to better present this paper and improve the narrative substantially, we would like to highlight to you that we totally reject reviewer 2’s criticism of our process. This reviewer likely has some subject expertise but seems to lack any methodological understanding of the construction of scoping reviews, where, in contrast, we claim academic expertise. Having re-read, consulted with independent colleagues, and considering reviewer 1’s feedback, we feel we were consistent and clear as to the research question and methodological approach in the original submission. Reviewer-2 suggests that our search strategy was incomplete. We refute this entirely and point out that our strategy was devised with subject and methodological experts, the details pre-recorded in a publicly available database, and that the search was completed and reported fully in line with the CONSORT guidelines. We provided all MESH terms and Boolean operators and the search parameters in the original submission. We note that reviewer-2 offers no suggestion as to where we have been deficient, merely a generic comment that they ‘think we have’. Perhaps tellingly as to the thoroughness of their review, the article they highlight as having been missed by our search was actually included in our original submission and is detailed in our results section, the tables and the references - being 1 of only 8 articles that we were able to include.

We are very pleased to be able add further context to our work. We apologise for the deficiencies in the writing of original manuscript and are grateful to the journals peer-review process for bringing this report up to scratch.

**Reviewers' comments:**
**Reviewer's Responses to Questions**

1. Is the manuscript technically sound, and do the data support the conclusions?

The manuscript must describe a technically sound piece of scientific research with data that supports the conclusions. Experiments must have been conducted rigorously, with appropriate controls, replication, and sample sizes. The conclusions must be drawn appropriately based on the data presented.

Reviewer #1: Yes

Reviewer #2: Partly

2. Has the statistical analysis been performed appropriately and rigorously?

Reviewer #1: Yes

Reviewer #2: I Don't Know

3. Have the authors made all data underlying the findings in their manuscript fully available?

The [PLOS Data policy](http://www.plosone.org/static/policies.action#sharing) requires authors to make all data underlying the findings described in their manuscript fully available without restriction, with rare exception (please refer to the Data Availability Statement in the manuscript PDF file). The data should be provided as part of the manuscript or its supporting information, or deposited to a public repository. For example, in addition to summary statistics, the data points behind means, medians and variance measures should be available. If there are restrictions on publicly sharing data—e.g. participant privacy or use of data from a third party—those must be specified.

Reviewer #1: Yes

Reviewer #2: Yes

4. Is the manuscript presented in an intelligible fashion and written in standard English?

PLOS ONE does not copyedit accepted manuscripts, so the language in submitted articles must be clear, correct, and unambiguous. Any typographical or grammatical errors should be corrected at revision, so please note any specific errors here.

Reviewer #1: Yes

Reviewer #2: No

5. Review Comments to the Author

Please use the space provided to explain your answers to the questions above. You may also include additional comments for the author, including concerns about dual publication, research ethics, or publication ethics. (Please upload your review as an attachment if it exceeds 20,000 characters)

6. PLOS authors have the option to publish the peer review history of their article ([what does this mean?](https://journals.plos.org/plosone/s/editorial-and-peer-review-process#loc-peer-review-history)). If published, this will include your full peer review and any attached files.

If you choose “no”, your identity will remain anonymous but your review may still be made public.

**Do you want your identity to be public for this peer review?** For information about this choice, including consent withdrawal, please see our [Privacy Policy](https://www.plos.org/privacy-policy).

Reviewer #1: **Yes:** Sahar Boozari

Reviewer #2: No

**Comments to Authors;**

**Reviewer #1:**

The care of brachial plexus injuries (BPI) in athletes, in my opinion, is a seriously under-discussed topic in sports medicine. By primarily relying on a small number of case studies for information, it successfully highlights the lack of comprehensive data and generally accepted best practices. This demonstrates the urgent requirement for more thorough research projects and established methods for diagnosis, assessment, and outcome reporting in BPI rehabilitation for sports-related injuries. however there are following issues about the manuscript:

**AUTHORS:**

Many thanks for taking the time to review our work. We are pleased that you agree with the importance and message of the findings.

**Reviewer:**
Abstract: The background section is notably concise and fails to provide substantial information about the context and significance of the study.

**AUTHORS:**

Thank you, Yes, in hindsight we agree that we’ve been much too concise and the introduction section was generally in need of a total rewrite. We agree that there was a need to sets the scene much better in sports BPI injury rehabilitation.

**Reviewer:**
Method: It's rather intriguing that, out of the numerous articles retrieved, only case reports met your inclusion criteria. Case reports generally constitute a lower level of evidence. Could you elaborate on the criteria used for selecting these reports while excluding others? Additional clarification on this aspect would be beneficial.

**AUTHORS:**

Yes, and it’s most disappointing. As you’ve noted, the purpose of our work was simply to try to find data relating to BPI rehabilitation and to report on these. We accepted any report containing primary data in our inclusion criteria - from RCT to case-report, irrespective of level of evidence. We looked far and wide, but this was all that there was. Mainly we found narrative pieces offering expert advice - but with no data. We are very confident in our technical search and that this is all the data that there is.

We have added further context around this to the methods, though we are of course limited in what we can do here around the specifics with reference to our original (pre-published) protocol. We report as per the PRISMA-ScR guidelines and have additionally provided the PCC framework which the search is based on as a table (as supplemental material). We also reaffirm in the first paragraph of our discussion that we looked for ALL data, and that this disappointingly poor data return was the major finding of this work.

**Reviewer:**
Consider adding a reference to define "contact sport."

**AUTHORS:**

Noted with thanks, reference provided in the amended manuscript submission.

**Reviewer:**
Result & Discussion:
While the section mentions variations in injury mechanisms and presentations, it does not delve into a detailed discussion of these variations or potential factors contributing to them.

The section mentions adverse events but does not provide comprehensive details or analysis of these events, which could be valuable for understanding potential risks associated with certain treatments.

**AUTHORS:**

Thanks also for these thoughts. While a sensible suggestion, we are anxious to delve too deeply into the detail of the presented case studies. As we only have 10 very specific examples of sports BPI injury and rehab, we are cautious as to the generalisability and representativeness of this data, and would prefer to keep to a more top-level discussion of the content provided. In additional to this general approach, the information provided in the case studies is often quite vague as to the injury mechanism. For example, we seldom get detailed information as to the phase of play, type of contact, severity of hit, kinematics at the time etc. Rather the cases seem to have been picked up at the rehab stage (often following a medical review) and the content written from there.

We have now detailed this in the revised discussion and think this addition helpful.

Notably the adverse events also don’t really relate to the rehab - which we are trying to keep the focus of our discussion on. The only significant AE relates to a surgical intervention.

Again, we have taken the opportunity to discuss this and we agree it a helpful addition.

**Reviewer #2:**

Dear authors
I have thoroughly reviewed your article on addressing deficiencies in the rehabilitation and clinical management of brachial plexus injuries in contact sports. I appreciate your efforts in this specific field. It is well-known that the incidence and severity of BPI varies based on the sport played. Depending on the degree of the injury's damage, the rehabilitation procedure varies. Many athletes return to sport without requiring postgame treatment.
Although the title of your study seems to be the rehabilitation of BPI, when I read the abstract and the entire article, I saw that your primary goal is to draw conclusions about the entire rehabilitation process and determine the criteria for return to sports. Despite the tight relationship between these two issues, they are distinct and must be addressed separately. If determining the clinical management and rehabilitation process is the primary objective, the chosen keywords are insufficiently specific. Because, on reviewing the entire article, I noticed that you included physiotherapy applications among the treatment methods. The study protocol that includes keywords also incorporates physiotherapy practices as keywords. Additionally, numerous other terms were used during the search. This indicates that the decision regarding keyword selection was inadequate. If the keywords are determined and chosen appropriately, you will be able to access more studies such as . S Reid et al.‘’ Brachial plexus injuries—report of two cases presenting to a sports medicine practice’’.

**AUTHORS:**

Dear reviewer, thanks for taking the time to return this peer review. We do appreciate the spirit of your comments, but are struggling to respond to some of this. We appreciate your helpful thoughts as to some our narrative, but you seem to have misinterpreted the more technical aspects of this work, and how the research question was addressed. We have written a rather lengthy response here, but hope that it sufficiently explains why many of the changes you suggest have not been enacted.

Yes, we absolutely agree that, colloquially, we know that there are various degrees of BPI related sports injury, that these manifest differently, are treated differently, and resolve differently. The purpose of this work though was to go beyond this colloquial clinical ‘knowledge’ and to look for the data that underpins rehabilitation management, and, were we able to find sufficient volume of this, we would ideally have tried to comment of the value of differential treatment strategies etc. to inform practice.

You suggest: “Although the title of your study seems to be the rehabilitation of BPI, when I read the abstract and the entire article, I saw that your primary goal is to draw conclusions about the entire rehabilitation process”. We feel you have misinterpreted us. We are looking at the rehabilitative management of contact sports-related BPI. To do this we searched for and extracted data as to rehabilitation management and also relevant contextual data around the injury history, presentation, diagnosis, diagnostics and outcomes.

We don’t understand the comment that you “saw our primary goal being about drawing conclusions about the entire rehabilitation process”. We clearly signposted this as a formal scoping review (applying correct and detailed scoping review methodological approaches as per the PRISMSA-ScR guidelines) to ‘systematically search and identify published peer-reviewed primary data relating to the clinical management guidelines of brachial plexus injuries in sport’. Further we specified that our intention was to ‘evaluate primary data on the rehabilitative management approaches, results, outcomes and return to play recommendations for BPI management in contact sport’ (phraseology drawn directly from our original submission; lines 9-11 and 91-93).

Your comments then as to “keywords being insufficient” really don’t make sense - or stand up to scrutiny. Review methodology relies on a well described process incorporating MeSH terms and Boolean operators within a defined search strategy in selected academic databases. We designed a detailed search strategy with a team of subject and methodological experts, supported by a subject specialist academic librarian, and published this prior to undertaking the review (as is best practice). The purpose of a scoping review is to cast a wide net and to filter down to the data that can answer the research question. We correctly apply broad MeSH terms to be as comprehensive as possible. The detailed search strategy (which we provided for review as supplemental material) highlights MeSH terms that encompass medical management, rehabilitation and return to play. This was essential to fully search all available data prior to our selection process, which is detailed in our PRIMSA flowchart. In light of your comments though we did invite an additional reviewer, with expertise in sports injury and literature review methodology, to review our search parameters and returned data. They have affirmed the appropriateness and comprehensiveness of our approach.

Specifically, our search protocol does NOT “also incorporate physiotherapy practices as keywords” as you incorrectly suggest. If you re-review the explicit search that we detail in the submission, you will see that the terms ‘physical therapy’ and ‘rehabilitation’ can be found, but nothing as to ‘treatment approaches or physiotherapy practices’.

We further contest your personal opinion that “keywords were chosen inadequately”. Rather, an appropriately qualified group constructed a thorough search strategy as per PRISMA-ScR guidelines criteria (which has now been independently reviewed and verified). We note you provide no suggestion as to any missing search parameters - merely a rather illiterate complaint. You suggest we would be “able to find more studies such as Reid and Trent 2002” with a more appropriate search. Embarrassingly, we already include this very study in our results and data tables. It is one of only eight papers that we were able to include following extensive searching, and perhaps casts doubt on the detail of your review of our technical processes.

**Reviewer:**

In the article's introduction, injuries are categorized as severe or moderate. What do the terms severe and moderate mean? What is the frequency of these types of injuries? Even if it is in separate sports branches, it is necessary to mention it in order to inform the reader. Expressions such as uncommon and frequent provide insufficient information to the reader.

**AUTHORS:**

Thank you, yes this is pertinent. We have totally rewritten the introduction section to provide further clarity to the reader. We now avoid problematic terms such as moderate or severe and instead focus on the degree of nerve damage. We also agree on the need to better contextualise injury frequency and have added context in terms of rate per 10,000 athlete exposures as is generally reported elsewhere.

**Reviewer:**
In paragraphs 79 and 80, is the phrase "In sports settings, BPIs are common enough for the slang terms'stinger' and 'burner' to be used?" a unique thought of the authors?

**AUTHORS:**

Thank you for highlighting this – the terminology is well established, but we have rephrased these lines as part of the rewrite of the introduction section and have added a reference as you suggest.

**Reviewer:**

Writing the introduction section in a more comprehensible, fluent and holistic manner will keep the reader's interest in the article alive.

**AUTHORS:**

Thank you. Point taken, we accept the need for a better introduction and address this in more detail in response to reviewer-1.

**Reviewer:**

In the imaging, diagnosis, and surgical interventions section, the RTP abbreviation was used without its meaning being specified. Similarly, table abbreviations should also be defined.

**AUTHORS:**

Thank you. Noted. We have amended this in the manuscript.

**Reviewer:**
Considering all of these circumstances
1- New and accurate keywords must be selected.
2- The purpose of the investigation must be determined thoroughly.
3- The introduction section should be written more clearly, fluently, and comprehensively.
4- The results and discussion sections should be rewritten based on the analysis of newly accessible articles.
I look forward to reviewing your article once these significant corrections have been made.

**AUTHORS:**

Thank you. We do appreciate your review, but feel that you have rather misinterpret our submission and the also the methodology underpinning scoping reviews. The search conducted is appropriate and technically correct for our stated purposes and all available data has already been incorporated. There are no new articles to add. We have though engaged with your critique of the narrative.

# 1. We dispute this – rationale provided.

# 2. We dispute this – rationale provided.

# 3. The introduction has been updated with thanks.

# 4. We dispute this – rationale provided.
