# Supplementary material for: Rehabilitation of brachial plexus injury in contact sport: Where are the data that underpin clinical management? A scoping review
Source: PLoS One. 2024 Jun 24;19(6):e0298317. doi: 10.1371/journal.pone.0298317 (PMC11195970; doi:10.1371/journal.pone.0298317)
Supplement: Attachment — Submitted filename: R2_BPI_Response to reviewers.docx [file pone.0298317.s005.docx]

**Response to reviewers**

Dear Dr Akaras and editorial team,

Please find enclosed our further revised manuscript entitled “Rehabilitation of brachial plexus injury in contact sport: Where are the data that underpin clinical management? A scoping review” which we re-submit for consideration of publication in PLOS ONE.

We are grateful to the reviewers for their additional minor comments and points for improvement, which we have acted on and updated the manuscript accordingly.

Best wishes

Author team

**Comments to the Author**

Review Comments to the Author

Reviewer #1:

The term "ambiguous" in relation to rehabilitative management (lines 120-122) needs a brief explanation for clarity. Further details or examples of local rehabilitation protocols mentioned in lines 118-122 would enhance the reader's understanding. The rationale behind excluding expert opinion articles should be briefly explained for transparency.

Reviewer #2: (No Response)

Authors comments:

Many thanks for highlighting these 3 points, we have addressed these and updated the enclosed manuscript version.

Many thanks for your constructive feedback to our paper.

Author team
